# Supplementary material for: Mitochondrial network complexity emerges from fission/fusion dynamics
Source: Sci Rep. 2018 Jan 10;8:363. doi: 10.1038/s41598-017-18351-5 (PMC5762699; doi:10.1038/s41598-017-18351-5)
Supplement: Supplementary file 1 — Supplementary Information [file 41598_2017_18351_MOESM1_ESM.pdf]

Supplementary Information for "Mitochondrial network complexity emerges from fission/fusion dynamics"

Zamponi N, Zamponi E, Billoni OV, Cannas SA, Helguera PR & Chialvo DR

Model supplementary information

We implemented the agent based model introduced by Sukhorukov *et al.* [1], namely, a set of L reactant objects corresponding to the network edges, submitted to the processes of fission and fusion dynamics through tip to tip and tip to side reactions of the type

$$2\,X_1 \rightleftharpoons X_2 \quad (1)$$

$$X_1 + X_2 \rightleftharpoons X_3 \quad (2)$$

where X_i $(i = 1, 2, 3)$ are the number of nodes with degree i.

Tip to tip reactions happen with association (dissociation) rate a_1 (b_1) between a random chosen pair of nodes with degree one (association) or for a random chosen site with degree 2 (dissociation). Tip to side reactions happen with association (dissociation) rate a_2 (b_2) between a random chosen pair of nodes with degrees one and two (association) or for a random chosen site with degree 3 (dissociation). Following Sukhorukov *et al.* [1], we assumed $b_2 = (3/2)b_1$ and varied the relative rates $c_i = a_i/b_i$.

Monte Carlo simulations were performed using Gillespie algorithm [3]. Most of the simulations were performed for $L = 15000$, the estimated average number of edges in the control cells images. Preliminary tests showed that the the number of nodes with degree k become stationary when the number of iterations is $\approx 2L$. So we run every simulation $3L$ iterations after which we measured different quantities. This procedure was repeated 100 times for different sequences of random numbers and the different quantities were averaged over this sample. The different quantities measured were: the average degree $\langle k \rangle$ (where the average is taken both over all the nodes in the network and over different runs), the average fraction of nodes in the largest cluster $\langle N_g/N \rangle$ (order parameter of the percolation transition) and the average cluster size excluding the largest cluster $\langle s \rangle$ (again the average is taken both over the network and over runs). $\langle s \rangle$ was calculated using the expression from classical percolation theory[2], namely if N_s is the number of clusters of size s and $n_s = N_s/N$, then

$$\langle s \rangle = \frac{\sum_s s^2 n_s}{\sum_s s n_s} \quad (3)$$

where the sums exclude the largest cluster in the network.

In Fig.1 we illustrate the typical behavior of the different quantities as a function of c_2 for a fixed value of c_1. As described in Ref.[1], for a given value of c_1, c_2 controls the percolation transition, characterized as a peak in $\langle s \rangle$, which happens when the order parameter (probability of a "giant component or cluster") is ≈ 0.3. Notice that the average degree is smaller than 2 around the critical region (onset of percolation). Such behavior was observed for all the range of values of c_1 of interest (i.e., those that correspond to values of the measured quantities observed in the experiments).

In Fig.2 we show a parametric plot of the order parameter vs. the average degree for a wide range of values of c_1. We see that all the curves intersect approximately at the same point, corresponding roughly to $\langle N_g/N \rangle = 0.8$ and $\langle k \rangle = 2$. Below those values, every point have associated well defined values of the parameters (c_1, c_2).

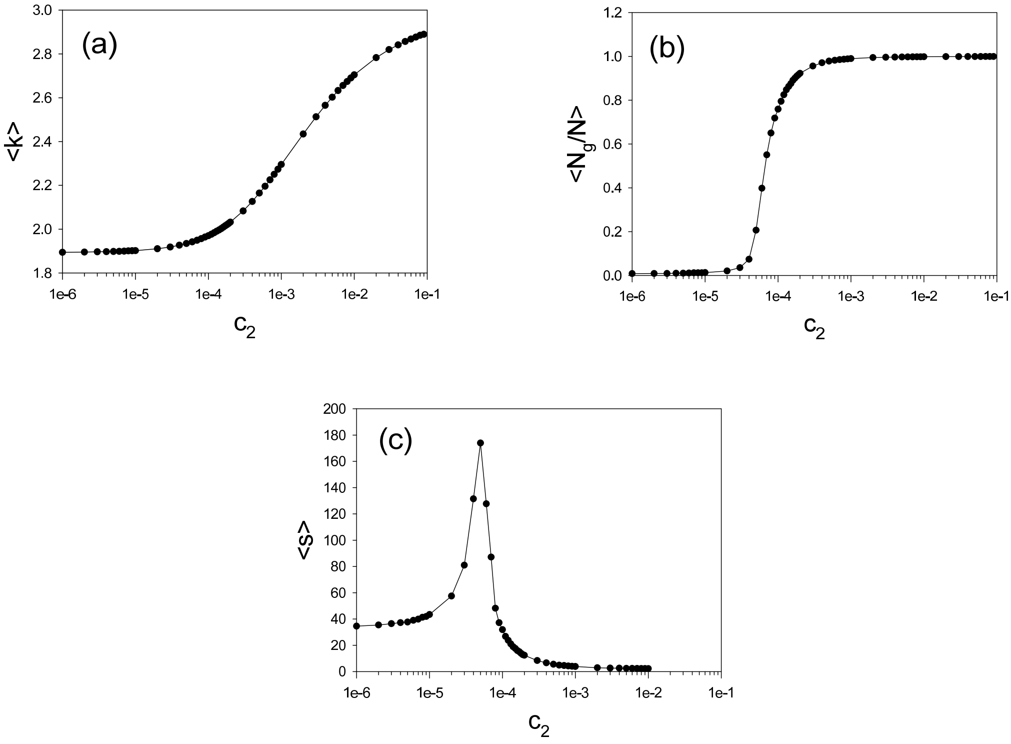

Figure 1: Monte Carlo results for $L = 15000$ and $c_1 = 0.01$. (a) Average degree. (b) Average fraction of sites in the largest ("giant") cluster. (c) Average clusters size.

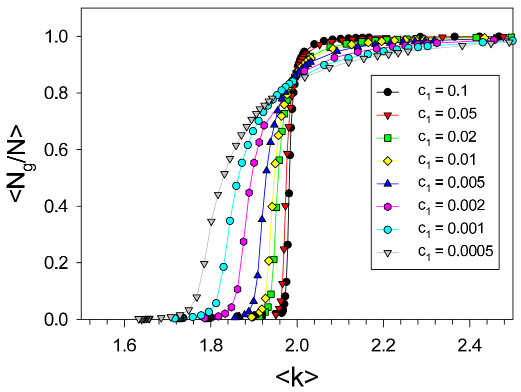

Figure 2: Order parameter vs. average degree for $L = 15000$ and different values of c_1.

Then, we compare the simulated curves of Fig.2 with the corresponding measured values in the experiments, as well as with their average for the three sets: CTL, PRQ and MFN. From this comparison we extracted the values of (c_1, c_2) for the three averages and compare them with the critical line in the (c_1, c_2) space, as shown in Fig. 5 of the main article. The critical line was obtained by tracking the values of c_2 at which the maximum of $\langle s \rangle$ is located for every value of c_1.

Experimental supplementary information

This section contains additional evidence supporting the main hypothesis as well as details about the network extraction procedure.

- The performance of the branching detection method is presented in Table 1. To evaluate the ability of our algorithm to detect branching points we selected multiple ROIs from confocal images corresponding to *ctl*, *pqt* and *mfn* treated cells (n=10). The number of true brances (*TP*) was obtained by visual inspection and constituted our gold standard set (*GS*). We then ran the algorithm on each ROI using three different thresholds ($th = 0.1$, 0.125 and 0.15) and extracted the predicted branching points in each case. Predicted branching points not present in the *GS* were counted false positives (FP). Conversely, branches present in the *GS* but absent in the predicted set were considered false negatives (FN). Using the described parameters, we computed the sensitivity of the procedure, defined as $s = \frac{TP}{TP+FN}$, and its precision, defined as $p = \frac{TP}{TP+FP}$. Values are presented in in Table 1

- It is possible that a 2D projection of the 3D mitochondrial structure might introduce an artificial superposition of network branches. The most probable artifact shall be the presence of nodes with degree four, byproduct of the intersection (in 2D) of linear segments. This issue was investigated by computing the number of degree four nodes in all conditions. Figure 3 contains the results of the calculations done to demonstrate that the frequency of the artifactual branching points is negligible.

- Figures 4 and 5 present data from additional experimental manipulations. BAPTA-AM, a cell-permeant Ca^{2+} chelator that boost ER-mediated mitochondrial fission, or FCCP, an uncoupling agent that cause mitochondrial membrane potential loss, were used to promote mitochondrial fission [4, 5]. Also, based on the fact mitochondrial structure rely on the cytoeskeleton, pharmacological modulation of actin filaments and microtubules was performed

th	sensitivity	precision
0.1	0.946 ± 0.024	0.851 ± 0.072
0.125	0.774 ± 0.04	0.836 ± 0.067
0.15	0.607 ± 0.071	0.732 ± 0.084

Table 1: *Performance of the branching detection procedure.* Sample ROIs from *ctl*, *pqt* and *mfn* images were analysed. Three different thresholds were used to convert images into skeletons. Sensitivity and precision were computed using $\frac{TP}{TP+FN}$ and $\frac{TP}{TP+FP}$, respectively.

by using Jasplakinolide or Taxol, respectively [6, 7, 8]. Finally, we over-expressed dynamin related protein 1 (DRP1), a pro-fission protein, or mitofusin 2 (MFN2), a pro-fusion protein.

Figure 4 presents examples of phenotypes obtained using each of the treatments and Figure 5 the structural analysis of the mitochondrial networks derived from those images. Notice that the results in Figure 5 are fully consistent with those presented in the main manuscript, confirming the generality of the conclusions obtained using paraquat treatment and mitofusin 1 over-expression.

- Each CCDF shown in Figure 6 was computed by selecting random sets of 5 networks of the corresponding condition. The result demonstrates that differences depicted in Figure 4 of the main manuscript are statistically significant.

References

[1] Sukhorukov, V.M., Dikov, D., Reichert, A.S., Meyer-Hermann, M. Emergence of the mitochondrial reticulum from fission and fusion dynamics. *PLoS Comput Biol* **8** e1002745 (2012).

[2] Barrat, A., Barthelemy, M., and Vespignani, A. Dynamical processes on complex networks. *Cambridge Univesity Press* (2008).

[3] Gillespie, D.T. Exact Stochastic Simulation of Coupled Chemical Reactions. *J. Phys. Chem.* **81** 2340-2361 (1977).

[4] Friedman, R.J., Lackner, L.L., West, M., DiBenedetto, J.R., Nunnari, J., Voeltz, G.K. ER Tubules Mark Sites of Mitochondrial Division. *Science* **334** 358-361 (2011).

[5] Li, S., Xu, S., Roelofs, B.A., Boyman, L., Lederer, W.J., Sesaki, H., Karbowski, M. Transient assembly of F-actin on the outer mitochondrial membrane contributes to mitochondrial fission. J Cell Biol **208** 109-123 (2015).

[6] Summerhayes, I.C., Wong, D., Chen, L.B. Effect of microtubules and intermediate filaments on mitochondrial distribution. *J Cell Sci* **61** 87-105 (1983).

[7] Morris, R.L., Hollenbeck, P.J. Axonal transport of mitochondria along microtubules and F-actin in living vertebrate neurons. *J Cell Biol* **131** 1315-1326 (1995).

[8] Drubin, D.G., Jones, H.D., Wertman, K.F. Actin structure and function: roles in mitochondrial organization and morphogenesis in budding yeast and identification of the phalloidin-binding site. *Mol Biol Cell* **12** 1277-1294 (1993).

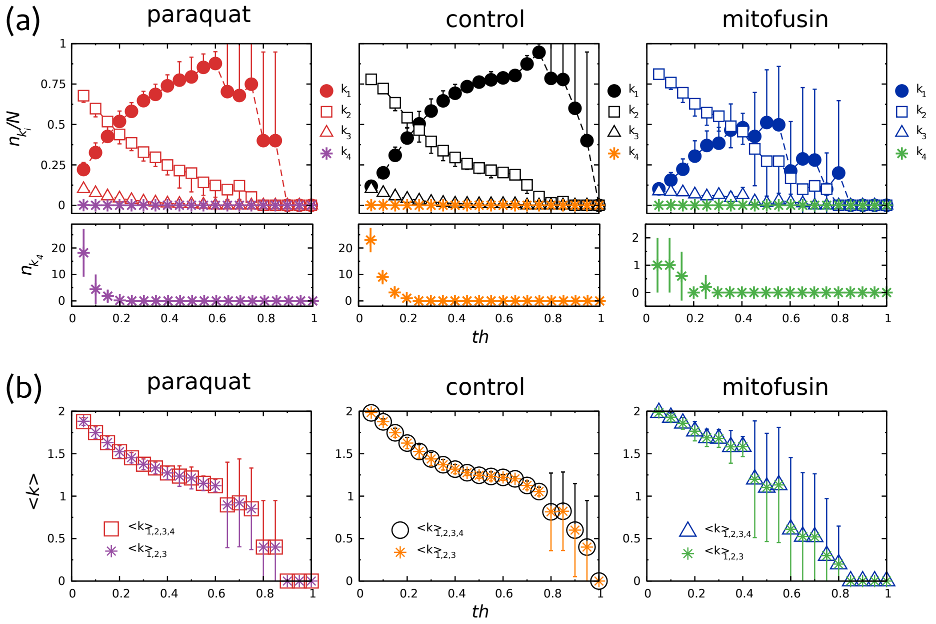

Figure 3: *Relative proportion of nodes of different degree and their contribution to mean degree ($\langle k \rangle$).* **(a)** Proportion of nodes (n_{k_i}/N, where N is the total number of nodes in the network and n_{k_i} is the number of nodes of degree i) with different degrees across conditions and thresholds (Upper panels). Although k_4 nodes are present in network reconstructions their frequency (Upper panels) and number (Lower panels) are low in every condition and at all thresholds. **(b)** Average $\langle k \rangle$ computed either including ($\langle k \rangle_{1,2,3,4}$) or excluding ($\langle k \rangle_{1,2,3}$) k_4 nodes. k_4 nodes do not significantly contribute to $\langle k \rangle$.

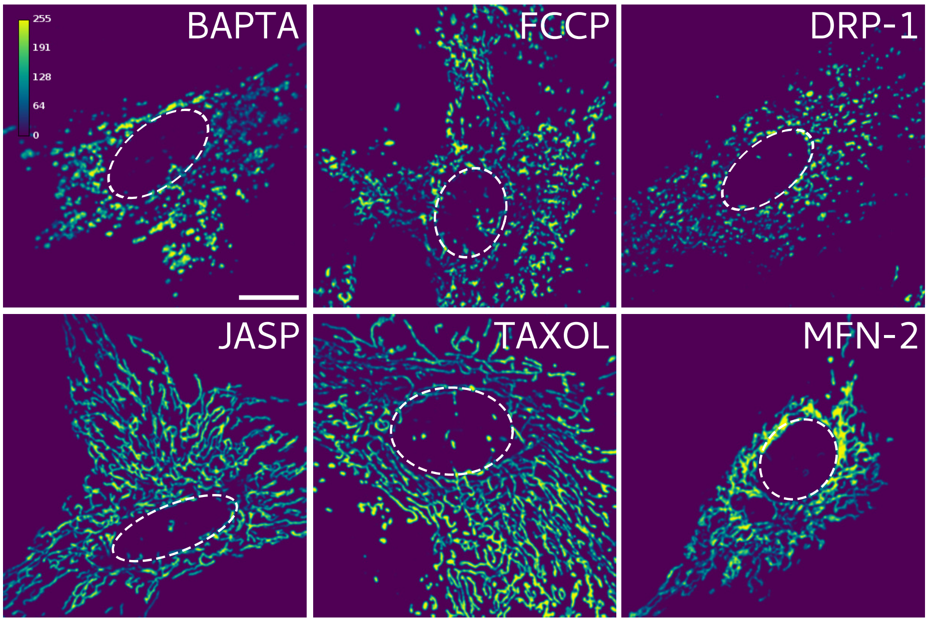

Figure 4: *Additional examples of mitochondrial network manipulations.* **Upper panel**: Mitochondrial network fragmentation induced by acute BAPTA-AM (10uM - 30min) or FCCP (20uM - 3h) treatment, or by dynamin-related protein 1 overexpression. **Lower panel**: Increased mitochondrial network fusion by Jasplakinolide (0.15uM - 30min) or Taxol (0.1uM - 30min) treatment, or by mitofusin 2 overexpression. Scale bar represents 10 μm.

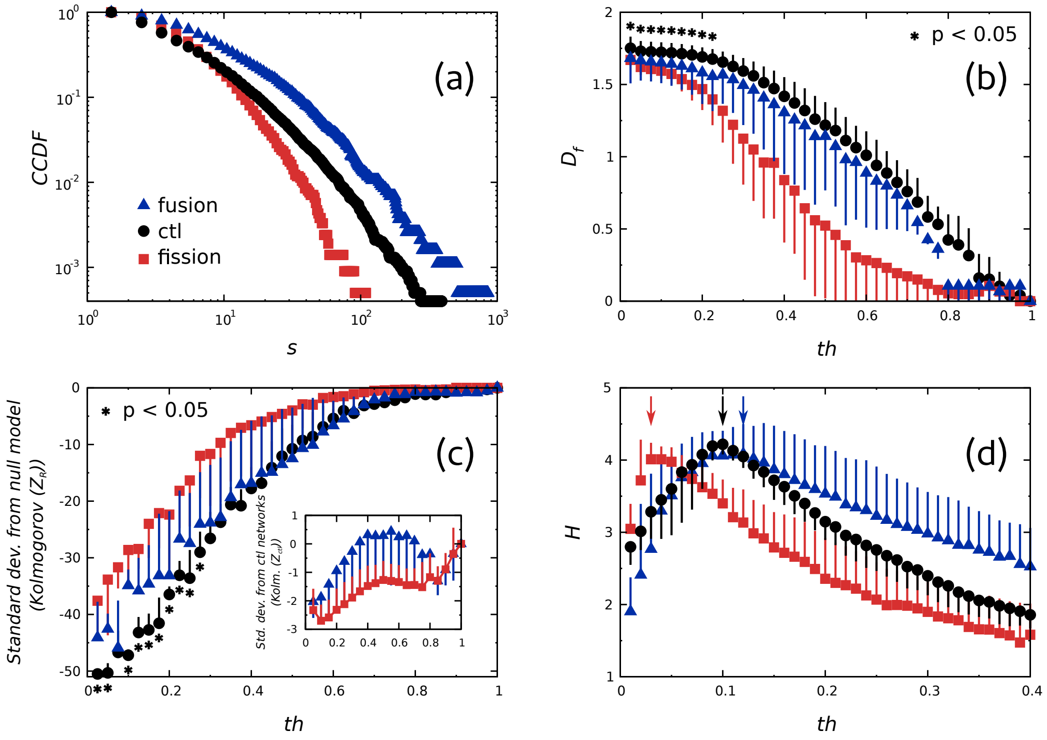

Figure 5: *Changes in mitochondrial network structural properties upon fission/fusion balance alterations.* Black circles correspond to control condition, while red squares and blue triangles correspond to drugs that promote mitochondrial fission and fusion, respectively. **(a)** Complementary cumulative distributions of cluster sizes. **(b)** Average fractal dimensions of the networks across the threshold range. Asterisks indicate statistically significant differences between ctl and treated networks ($p < 0.05$). **(c)** The deviation from a random structure was calculated using Kolmogorov complexity. Asterisks indicate statistically significant differences between ctl and treated networks ($p < 0.05$). Inset panel shows the deviation from the control structure of fissioned and fused networks. **(d)** Shanon entropy of cluster mass distributions. Shifts in the critical threshold th^* upon treatment are depicted (red and blue arrows).

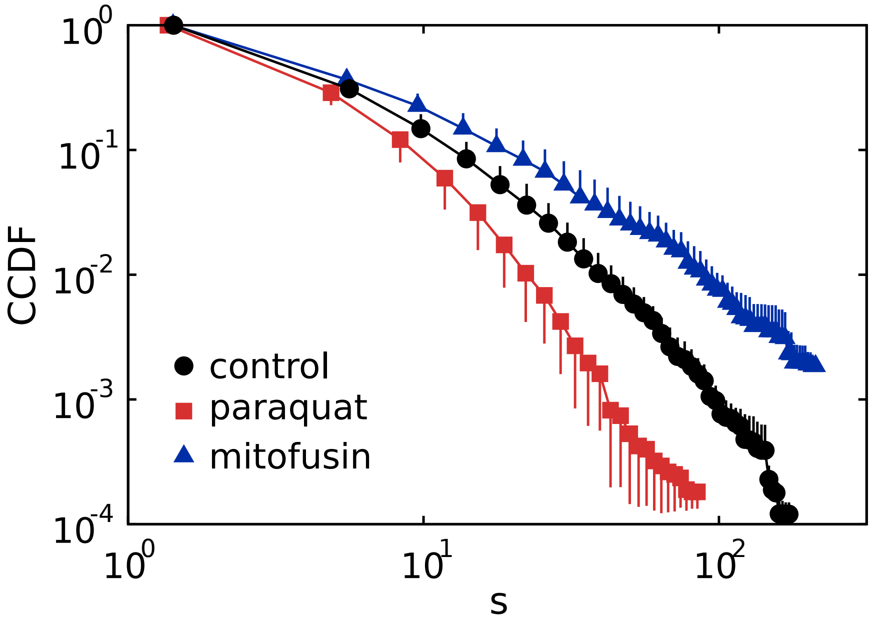

Figure 6: *Average behavior of cumulative cluster size distributions of control and perturbed networks.* A random permutation procedure was implemented to calculate the average cumulative distribution of cluster sizes for sets of 5 networks. Within each group, every combination of 5 out of 10 networks was used to build the average distributions.
